# *btn1* Affects Endocytosis, Polarization of Sterol-Rich Membrane Domains and Polarized Growth in *Schizosaccharomyces pombe*

**DOI:** 10.1111/j.1600-0854.2008.00735.x

**Published:** 2008-06

**Authors:** Sandra Codlin, Rebecca L Haines, Sara E Mole

**Affiliations:** 1 MRC Laboratory for Molecular Cell Biology, University College LondonWC1E 6BT London, UK; 2 Department of Biology, University College LondonWC1E 6BT London, UK; 3 General and Adolescent Paediatric Unit, UCL Institute of Child Health, University College LondonWC1N 1EH London, UK

**Keywords:** Batten, *btn1*, *CLN3*, *end4*, endocytosis, F-actin, *myo1*, neuronal ceroid lipofuscinosis, polarity, *sla2*, sterol, *Sz. pombe*

## Abstract

*btn1*, the *Schizosaccharomyces pombe* orthologue of the human Batten disease gene *CLN3*, exerts multiple cellular effects. As well as a role in vacuole pH homoeostasis, we now show that Btn1p is essential for growth at high temperatures. Its absence results in progressive defects at 37°C that culminate in total depolarized growth and cell lysis. These defects are preceded by a progressive failure to correctly polarize sterol-rich domains after cytokinesis and are accompanied by loss of Myo1p localization. Furthermore, we found that in *Sz. pombe*, sterol spreading is linked to defective formation/polarization of F-actin patches and disruption of endocytosis and that these processes are aberrant in *btn1*Δ cells. Consistent with a role for Btn1p in polarized growth, Btn1p has an altered location at 37°C and is retained in actin-dependent endomembrane structures near the cell poles or septum.

Juvenile-onset neuronal ceroid lipofuscinosis (JNCL) or Batten disease, a severe neurodegenerative lysosomal storage disease of childhood characterized by accumulation of lipofuscin-like material, is caused by mutations in *CLN3*. JNCL presents between the ages of 6–10 years with visual failure, followed by epilepsy and progressive cognitive and motor deterioration, leading to premature death. The conservation of *CLN3* suggests that it has a basic cellular role, although its exact function is still unknown. CLN3 is a transmembrane protein (1–5) and is located in the endosome–lysosome membrane and possibly in lipid rafts (6,7). Its function has been linked to many cellular processes, including trafficking 8), cytoskeletal organization 9), lysosomal homoeostasis (10,11), autophagy 12), apoptosis 13) and lipid modification or changes (14,15).

To help understand the apparently complex role of CLN3, we have been using a simple model system, the fission yeast *Schizosaccharomyces pombe*. We have previously shown that deletion of *btn1*, the *CLN3* orthologue, causes dysregulation of vacuole homoeostasis, with *btn1*Δ cells displaying larger and less acidic vacuoles (equivalent to mammalian lysosomes) 16), as in patient cells 10). In addition, we demonstrated that in *Sz. pombe*, Btn1p traffics slowly to the vacuole membrane through the endosomal system and Btn1p has a role prior to reaching the vacuole that impacts on vacuole function. Ectopic expression of N-terminally fused green fluorescent protein (GFP)–Btn1p or GFP–CLN3 constructs in *btn1*Δ cells complements the vacuole size and pH defects, confirming that Btn1p and CLN3 are functional homologues.

We also found that in the absence of a functional V-type H^+^ adenosine triphosphatase (v-ATPase), *btn1* is required for completion of cytokinesis 16), suggesting an additional role for *btn1* that is not linked to vacuole pH regulation. Consistently, we noted that at 25°C, under normal growth conditions, cells deleted for *btn1* have an increased septation index, are longer and grow somewhat slower than wild-type cells 16). Given the tight control of progression through the cell cycle in fission yeast, we reasoned that the mild growth defects of *btn1*Δ cells could be significant and so examined the phenotype of these cells under different growth conditions. We discovered that *btn1*Δ cells are sensitive to growth at high temperature and lyse at 37°C following progressive depolarization of growth. Subsequent analysis revealed that at 37°C, Btn1p is involved in an F-actin-dependent process that links endocytosis and the polarized localization of sterol-rich membrane domains with polarized growth.

## Results

### Btn1p is essential for growth at 37°C

**Figure 1:**
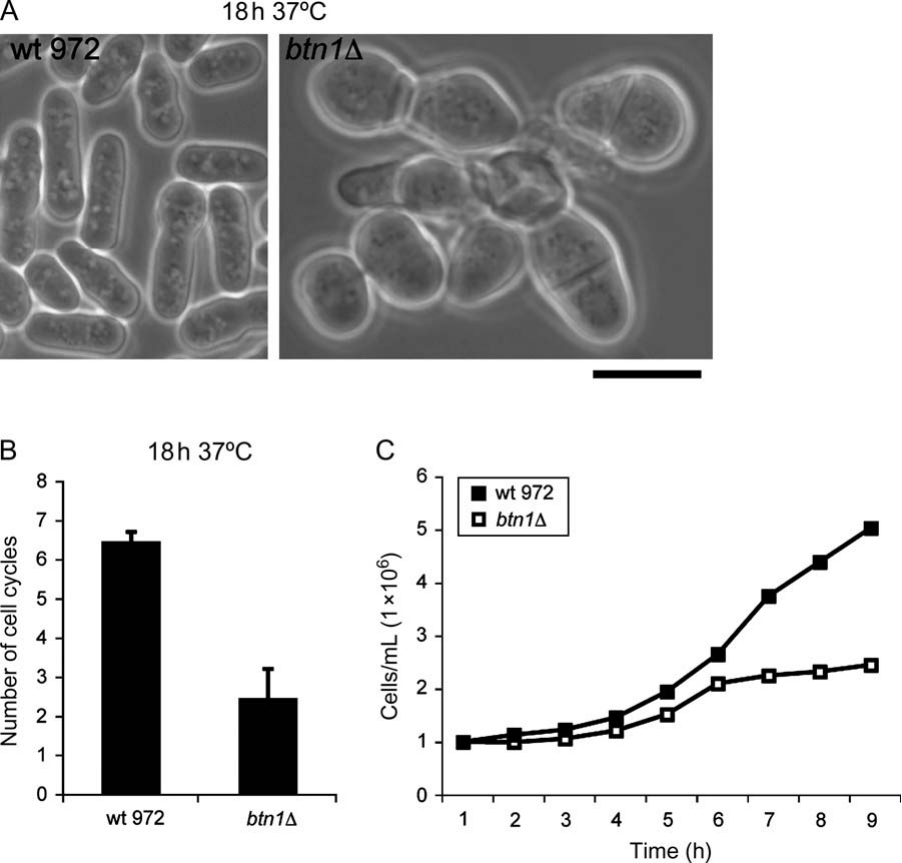
***btn1*Δ cells are temperature sensitive for growth at 37°C.**A) Phase contrast of wild-type 972 and *btn1*Δ cells grown for 18 h at 37°C. B) Graph of number of cell cycles completed in 18 h in YES at 37°C, as determined by cell counting (three replicates). C) Growth curve of wild-type 972 compared with *btn1*Δ cells in 9 h following a temperature shift from 25°C to 37°C. Scale bar, 10 μm.

We explored the effect of increased temperature on the growth of *btn1*Δ cells. The presence of *btn1* is not essential for growth of fission yeast cells under normal growth conditions. However, at 37°C in YES media, *btn1*Δ cells were found to be temperature sensitive for growth. After overnight growth (18 h) at 37°C, *btn1*Δ cells underwent striking changes in cell morphology, with the appearance of swollen cells that were pear shaped, elliptical or branched, and this was accompanied by some cell lysis (Figure 1A). *btn1* Δ cells progressed through no more than two to three cell cycles within this time compared with at least six cell cycles for wild-type cells as measured by cell count (Figure 1B) and time lapse microscopy (data not shown). By 24–48 h at 37°C, all *btn1*Δ cells had undergone lysis. Growth curve analysis of early events at 37°C (Figure 1C) revealed that after transfer to 37°C, both wild-type and *btn1*Δ cells underwent a delay in growth, presumably because of activation of the heat-stress response pathway 17), but by 3 h growth had resumed in both strains. However, unlike wild-type cells, which appeared to have completed the first cell cycle by 5 h, *btn1*Δ cells were slightly delayed and took 6–7 h to reach this stage. After this point, growth was significantly delayed for *btn1*Δ cells compared with wild-type cells (Figure 1C). Thus, *btn1*Δ cells display severe and progressive growth defects at 37°C.

### btn1Δ cells are defective in the distribution of sterol-rich membrane domains

**Figure 2:**
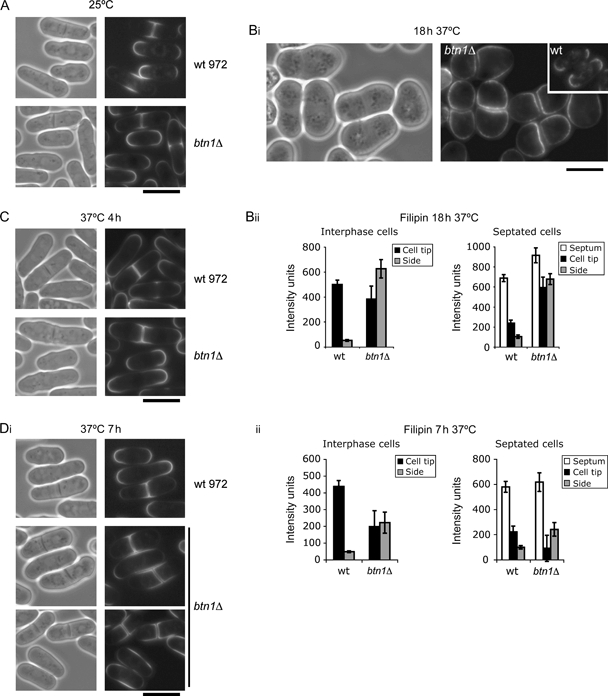
***btn1*Δ cells have altered sterol-rich membrane domains.**Loss of polarization of sterol domains in *btn1*Δ cells at 37°C: filipin staining of wild-type 972 and *btn1*Δ cells grown at A) 25°C and Bi) 37°C for 18 h (left panel, phase contrast; inset, wild-type 972). Bii) Spatial distribution graphs of filipin density of indicated strains grown at 37°C for 18 h for interphase and septated cells, *n* = 25. Onset of filipin staining is linked to completion of cytokinesis: filipin staining of wild-type 972 and *btn1*Δ cells grown at 37°C for C) 4 h and Di) 7 h. Dii) Spatial distribution graphs of filipin density of indicated strains grown at 37°C for 7 h for interphase and septated cells, *n* = 25 individual measurements. Scale bar, 10 μm.

The changes in cell shape are consistent with the loss of polarized growth. In fission yeast, sites of polarized growth and cell wall deposition are restricted to the cell poles in interphase cells and to the mid-zone, the site of cell division, in dividing cells. These sites form at and are maintained by the presence of sterol-rich membrane domains (lipid rafts) 18) that can be visualized by staining with filipin, a fluorescent sterol-binding dye. We examined the distribution of filipin-stained sterol domains in cells grown at 25°C and 37°C. These domains were correctly polarized at sites of growth in wild-type cells and in cells lacking *btn1* grown at 25°C (Figure 2A). However, in contrast to wild-type cells that retained polarized filipin up to and after 18 h growth at 37°C, in *btn1*Δ cells, the polarization of filipin-stained sterol domains was lost (Figure 2B) and instead, these domains were depolarized and distributed over the plasma membrane surface. Spatial distribution graphs of filipin staining confirmed that in *btn1*Δ cells, there was increased filipin staining along the sides of interphase cells and at all locations in septated cells, particularly at the septa and cell sides (Figure 2C). Thus, in *btn1*Δ cells, after 18 h at 37°C, sterols are often concentrated at swollen regions of the cell including the division septum, suggesting that these cells are unable to correctly delimit sterols to cell poles during interphase or during the final stages of cell division after prolonged growth at 37°C. This broadening of filipin staining is consistent with the depolarized growth of *btn1*Δ cells. In order to establish whether sterol spreading was merely a secondary consequence of depolarized growth, we examined the location of these sterol-rich domains in *btn1*Δ cells at earlier time-points after the temperature shift to 37°C. Up to and including 4 h at 37°C, their location was correctly polarized in both wild-type cells and *btn1*Δ cells (data not shown and Figure 2C), but by 7 h at 37°C, in *btn1*Δ cells, sterol spreading began to occur. Interphase cells displayed broad regions of filipin staining either on the cell sides or spreading from the cell tips, and dividing cells showed sterol spreading from septum regions (Figure 2D). Spatial distribution graphs confirmed these findings. Additionally, in septated cells, filipin staining was observed mainly spreading outwards from the septum regions on to the cell sides but was rarely at the cell tips. At this time-point, *btn1*Δ cells are still largely rod shaped and not yet completely depolarized for growth. Thus, progressive spreading of sterols in *btn1*Δ cells at 37°C appears to precede total depolarized growth.

### Btn1p affects the localization of Myo1p at 37°C

**Figure 3:**
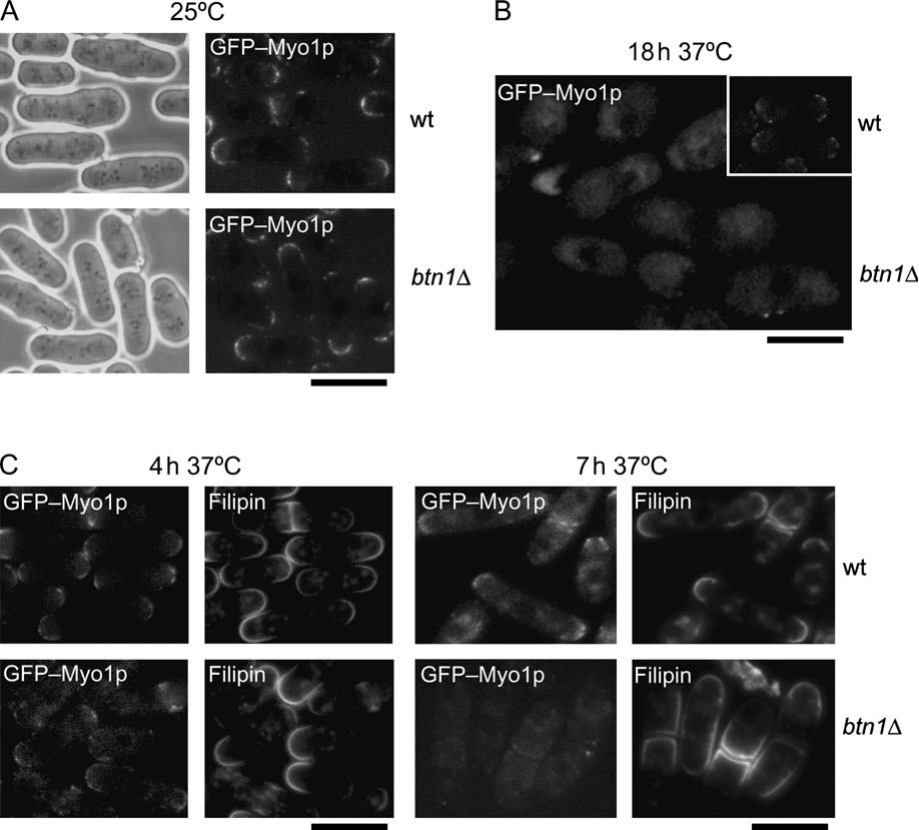
***btn1*Δ cells have altered Myo1p location.**Loss of polarization of sterol domains is linked to altered localization of GFP–Myo1p in *btn1*Δ cells at 37°C: localization of GFP–Myo1p in wild-type and *btn1*Δ cells at A) 25°C and B) 37°C for 18 h (inset, wild type). C) Altered localization of GFP–Myo1p precedes total depolarization of growth in *btn1*Δ cells at 37°C: localization of GFP–Myo1p in wild-type and *btn1*Δ cells at 4 h and at 7 h at 37°C with filipin staining. Scale bar, 10 μm.

In *Sz. pombe*, polarization of sterol-rich membrane domains has been shown to be dependent on *myo1*, which encodes a type 1 myosin motor protein 19). We therefore explored whether localization of Myo1p was affected in *btn1*Δ cells. We crossed *btn1*Δ with a strain carrying endogenous Myo1p fused with GFP and found that although at 25°C Myo1p localized to cell tips and septum regions in both wild-type and *btn1*Δ cells (Figure 3A), after 18 h at 37°C, the location of Myo1p was completely depolarized in *btn1*Δ cells, showing mainly a diffuse or occasionally a depolarized punctate pattern of staining (Figure 3B). This depolarization concurred with the previously observed spreading of filipin staining (Figure 2B). We also observed Myo1p and filipin location at earlier time-points during growth at 37°C, prior to the cell swelling phenotype. Myo1p and sterol-rich domains were correctly polarized and found at the cell tips of interphase cells and at septa of dividing cells up to and including 4 h of growth at 37°C (Figure 3C). However, by 7 h at 37°C, GFP–Myo1p was fully depolarized in *btn1*Δ cells (Figure 3C). This coincided with the onset of filipin spreading (Figure 3C). In contrast, polarized location of Myo1p and sterols was observed in wild-type cells expressing GFP–Myo1p and grown under the same conditions (Figure 3B,C). Thus, mislocalization of Myo1p occurs with onset of sterol spreading and precedes total depolarized growth in *btn1*Δ cells, which occurs at 7 h at 37°C. These events are concurrent with completion of the first cell cycle for the total cell population (Figure 1C). Together, these data suggest that polarization of growth zones is defective post-cytokinesis in *btn1*Δ cells at high temperature.

### Polarization of sterol-rich domains is linked to the correct formation and polarization of F-actin patches and endocytosis

We explored the mechanism underlying Myo1p loss and its link to sterol spreading in more detail. Myo1p, the only type 1 myosin in fission yeast and its *Saccharomyces cerevisiae* orthologues, Myo3p and Myo5p, have independently been shown to be directly involved in the activation of the Arp2/3 complex at the plasma membrane. Arp2/3 activation promotes actin polymerization and drives F-actin patch formation at these sites (20–26). Deletion of genes encoding components of the Arp2/3 complex in *Sz. pombe* is lethal (27,28), but deletion of *myo1* largely results in defective formation and depolarization of F-actin patches 20) consistent with the observed depolarized growth phenotypes.

**Figure 4:**
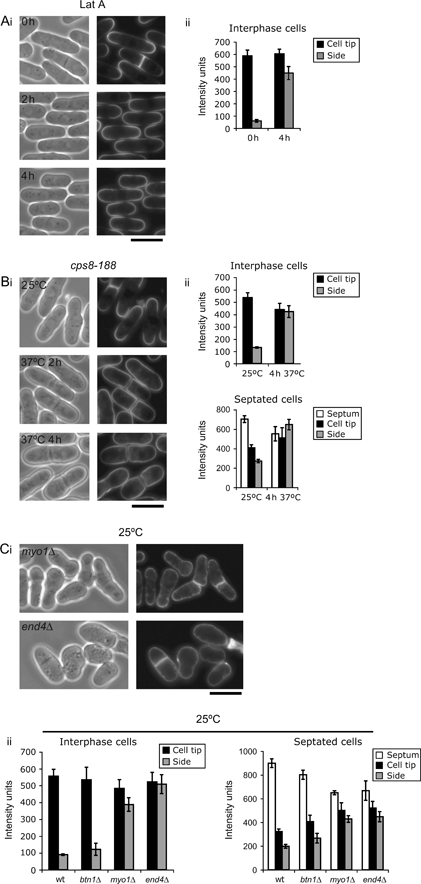
**Polarization of sterol-rich domains, F-actin patch formation and polarization and endocytosis are linked.**Loss of polarisation of sterol domains is linked to defects in F-actin patch formation/polarisation: Ai) filipin staining of wild-type cells treated with Latrunculin A (Lat A) for indicated times and ii) spatial distribution graph of filipin density of those cells that were in interphase; Bi) filipin staining of the actin *ts* mutant strain *cps8-188* grown at 25°C and 37°C for indicated times and ii) spatial distribution graphs of filipin density of interphase and septated strains grown at 37°C for 4 h. Loss of polarisation of sterol domains is linked to defects in endocytosis: Ci) filipin staining of F-actin patch mutant *myo1*Δ and endocytic mutant *end4*Δ grown at 25°C and ii) spatial distribution graph of filipin density of indicated cells in interphase and septation, *n* = 25 individual measurements. Scale bar, 10 μm.

We tested whether sterol spreading is linked to depolarization of the F-actin patch machinery. First, we examined the location and spreading of sterol-rich domains in cells totally defective in F-actin polymerization by the use of Latrunculin A, which disrupts the F-actin cytoskeleton by sequestering monomeric actin. At the concentration used, all F-actin cables and patches depolymerize by 10 min (Figure S1). We found that polarized sterol domains are not maintained after prolonged Latrunculin A treatment and filipin is instead spread over most of the cell surface in all cells after 2–4 h of treatment (Figure 4A), with the exception of small regions adjacent to the nuclei that remain free from sterol spreading. We measured filipin density in interphase cells (i.e. cells with one nucleus), which confirmed sterol spreading along the sides of the cells. Latrunculin A treatment prevents cell cycle progression to cytokinesis because the acto–myosin ring is not formed and the division plane is not assembled; so, we were unable to measure filipin density in septated cells. A previous report 19) describes broad bands of sterols on cell sides in *cdc25-22*‘block and release’ cells that had been treated with Latrunculin A. The authors suggest that this treatment allows the proportion of cells that proceed through mitosis to attempt assembly of a sterol band at the cell sides.

Next, we tested whether sterol spreading occurred in an actin mutant strain (*cps8-188*) 29) when grown at the non-permissive temperature. These cells exhibit a block at septation and depolarized growth consistent with a severe defect in actin after 2–4 h growth at 37°C. Again, severe sterol spreading was observed after 2 h at 37°C that worsened by 4-h growth, by which time the depolarized phenotype was apparent (Figure 4B). Spatial distribution graphs of filipin spreading confirmed this finding with sterol spreading to cell sides apparent in both interphase and septated cells (Figure 4B). These data support the hypothesis that polarization of F-actin and localization of sterol domains are linked.

We then explored the link between sterols and F-actin patches. Investigations in both *S. cerevisiae* and *Sz. pombe* yeasts have recently provided a link between F-actin patch formation and polarization and endocytosis. Indeed, ‘slow-moving’ F-actin patches at the plasma membrane have been identified as sites of endocytic membrane invagination (reviewed in 30), and the active process of actin polymerization at these sites forms part of the Sla1p and Sla2p/End4p-dependent endocytic machinery that causes the membrane to invaginate, ultimately giving rise to an early endocytic vesicle. F-actin patch formation at the plasma membrane is therefore not only coupled to endocytosis but also part of the endocytic process. Consistently, type 1 myosin has also been linked to endocytosis in *S. cerevisiae*(24,26,31), although not apparently in *Sz. pombe*^19^).

**Figure 5:**
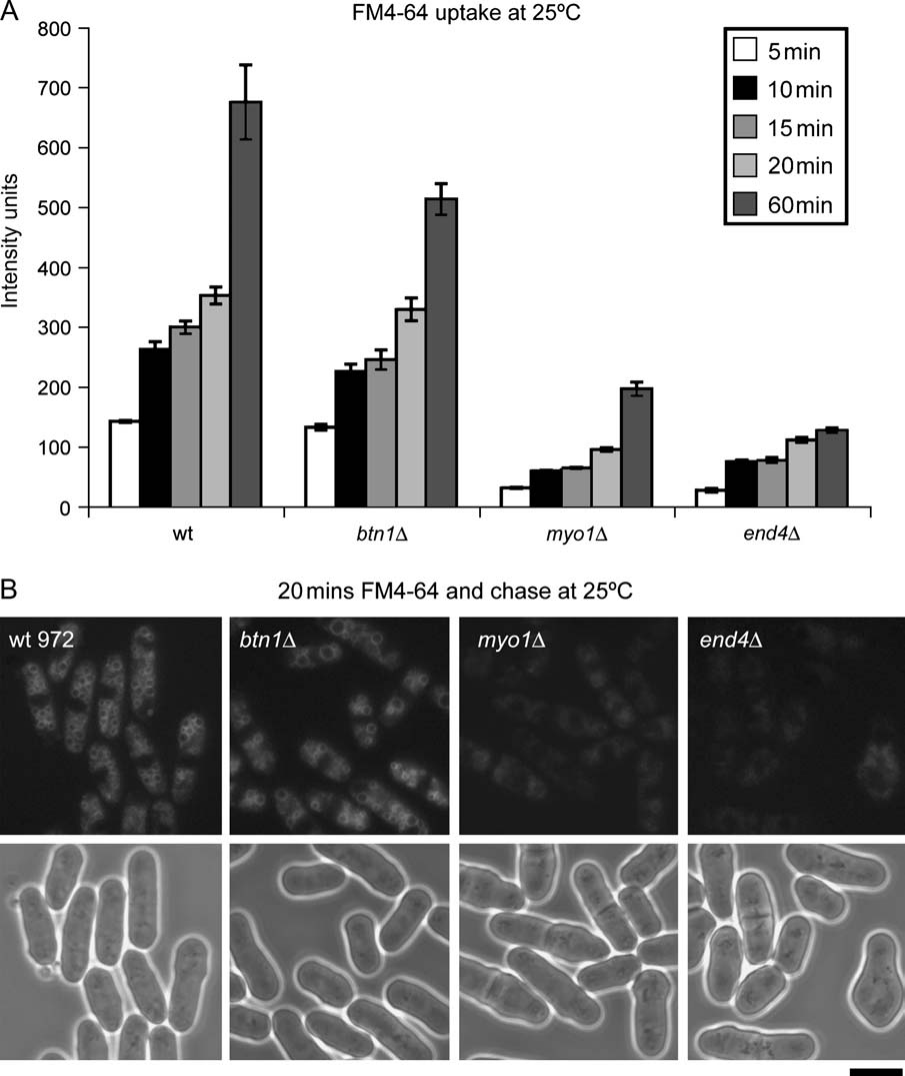
***btn1*Δ, *end4*Δ and *myo1*Δ cells grown at 25°C are defective in endocytosis.**At 25°C, *btn1*Δ cells are slightly defective, and *end4*Δ and *myo1*Δ cells are severely defective for endocytosis. A) Graph of FM4-64 uptake in intensity units over time of indicated strains. B) Uptake of FM4-64 after 20 min of incubation plus chase of indicated cells grown at 25°C. Scale bar, 10 μm.

We tested cells that were compromised in early stages of endocytosis and/or F-actin patch polarization for sterol spreading. The *sla2*/*end4*Δ strain is defective in the membrane internalization step of endocytosis 32) and consistently displays severe F-actin defects (depolarized F-actin patches) and depolarized growth. The *myo1*Δ strain is also defective for F-actin patch formation/polarization 20). We found that sterols spread over the entire cell surface in *end4*Δ cells even at the permissive temperature of 25°C (Figure 5C). We also confirmed sterol spreading in *myo1*Δ cells at this temperature [Figure 4C and as previously reported 19)]. Measurement of the filipin intensity over the cell surface in interphase and septated cells confirmed that sterol spreading occurs in both cell types (Figure 4C). We noted that this was more severe in *end4*Δ cells compared with *myo1*Δ cells (Figure 4C), particularly at interphase, which may underlie the more severe defect in polarized growth in this strain.

We went on to investigate whether sterol spreading was directly linked to endocytic defects in these strains. Endocytic defects are typically visualized by monitoring the uptake and trafficking of the lipophilic dye FM4-64, which is taken up at the cell tips in interphase cells and septum region in dividing cells 33) and transported to the vacuole within minutes. We found that at 25°C, cells deleted for *end4*, already known to have a severe endocytic defect 32), show very limited uptake of FM4-64 over time compared with wild-type and *btn1*Δ cells [Figure 5A,B and 32)]. We also found that *myo1*Δ cells are severely compromised for endocytosis, with minimal uptake of FM4-64 over time (Figure 5A,B). Even after 60-min incubation, only very low amounts of FM4-64 had entered *myo1*Δ cells. FM4-64 has been observed to eventually traffic to the vacuole (19,32), albeit at very reduced amounts, in these strains (Figure 5B). Although it has been reported that the *myo1*Δ strain does not have a defect in endocytosis 19), we show, using a more sensitive method of FM4-64 uptake, that this same strain is in fact severely delayed in endocytosis. When we used the previously reported method where excess FM4-64 is preloaded onto cells on ice prior to uptake 19), we found that although FM4-64 is ultimately taken up (between 10 and 20 min), at earlier time-points (1–10 min), a delay was clearly evident (Figure S2). The method used in the previous report 19) is more suited to detection of a total block in endocytosis rather than a delay unless earlier time-points are examined. We concluded that there is a link between defective F-actin patch polarization, defective endocytosis and sterol domain spreading.

### btn1Δ cells are defective in F-actin patch formation and polarization

**Figure 6:**
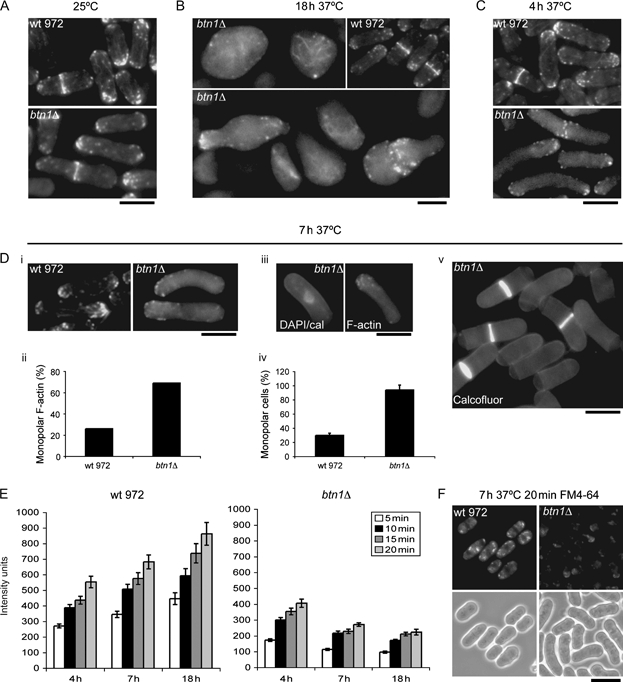
***btn1*Δ cells are defective in F-actin patch formation/polarisation and endocytosis.** *btn1* Δ cells have slight defects in the F-actin cytoskeleton at 25°C. A) Rhodamine–phalloidin actin staining of wild-type 972 and *btn1*Δ cells grown at 25°C. *btn1* Δ cells develop severe defects in the F-actin cytoskeleton at 37°C. B) Loss of polarisation of F-actin patches in *btn1*Δ cells grown for 18 h at 37°C (inset, wild-type 972 grown under same conditions). C) Actin staining of wild-type and *btn1*Δ cells grown for 4 h at 37°C. Monopolar F-actin patch localization causes NETO defects in *btn1*Δ cells prior to total depolarized growth at 37°C. Di) Rhodamine–phalloidin actin staining of wild-type 972 and *btn1*Δ cells grown for 7 h at 37°C, ii) graph showing the percentage of indicated interphase cells with monopolar (rather than bipolar) actin (*n* = 300), iii) calcofluor/DAPI (left) and rhodamine–phalloidin F-actin (right) staining of *btn1*Δ cells grown for 7 h at 37°C, iv) graph showing the percentage of indicated cells monopolar for growth (by calcofluor staining) (three replicates of *n* = 300) and v) calcofluor staining of *btn1*Δ cells grown for 7 h at 37°C showing monopolar growth caps. *btn1* Δ cells are severely defective for endocytosis at 37°C. E) Graph of FM4-64 uptake of wild-type and *btn1*Δ cells grown for indicated times at 37°C, displayed in intensity units over time. F) Panel showing 20 min FM4-64 uptake of indicated cells grown at 37°C for 7 h. Scale bar, 10 μm.

Because *btn1*Δ cells are defective in sterol localization at 37°C and we have now demonstrated a link between sterol spreading, depolarization of F-actin and reduced endocytosis, we examined *btn1*Δ cells for such defects. First, we stained for F-actin in *btn1*Δ cells grown at 25°C and 37°C. At 25°C, the distribution of F-actin in *btn1*Δ cells appeared relatively normal with polarized F-actin patches at the poles in interphase cells and at the septum in dividing cells (Figure 6A). We did however note that there was more diffuse F-actin in these cells compared with wild-type cells. At 37°C, after 18 h, by which time *btn1*Δ cells were already depolarized for growth (Figure 1A), severe F-actin defects were apparent. F-actin patches were mostly depolarized or completely absent (Figure 6B), with only a few cells displaying some polarized F-actin patches that were found at the polarized (non-swollen) end of pear-shaped cells. We explored earlier time-points at 37°C to follow the course of F-actin depolarization and its link to sterol spreading. At 4 h at 37°C, F-actin patches were mainly polarized in both wild-type and *btn1*Δ cells (Figure 6C), although again we noted more diffuse F-actin in *btn1*Δ cells. However, at 7 h at 37°C, *btn1*Δ cells exhibited a very diffuse F-actin staining together with defective localization of F-actin patches (Figure 6D). The majority of *btn1*Δ cells (71%, compared with 26% of wild-type cells) displayed a monopolar, rather than bipolar, distribution. For those patches localized at, or near, the one growing tip, as evidenced by calcofluor staining (Figure 6D), the distribution was not centred at the tip as in wild-type cells. Rather, patches were more dispersed around the tip. The predominance of monopolar over bipolar F-actin patches at 7 h suggested a defect in maintaining bipolar growth. This is likely to be a prerequisite for the more severe polarity defects at later time-points.

*Sz. pombe* cells typically grow in a bipolar manner, that is, at both cell tips. Briefly, following cell division, each daughter cell first resumes polarized growth at the ‘old’ end, marked by the polarized presence of F-actin patches, and thus grows in a monopolar manner for a set time. At a later point in G2, a ‘new end take-off’ (NETO) pathway 34) is established that additionally localizes F-actin at the new cell end, enabling polarized growth at this end as well as the old end (bipolar growth) until cell division. Cells that are blocked in NETO 35) continue to grow but remain monopolar in their growth up to septation. We therefore determined the percentage of septated cells that had only one growth cap, as visualized by calcofluor staining after 7 h growth at 37°C. Ninety-four per cent of septated *btn1*Δ cells were monopolar for growth at this time compared with 30% of wild-type cells (Figure 6D). Thus, by 7 h, after completion of the first cell division at 37°C and preceding the swollen growth phenotype, *btn1*Δ cells have a defect in polarizing F-actin, particularly at a new growth zone, that prevents the initiation of polarized growth at a new cell end. Further analysis revealed that after 7 h, the F-actin cytoskeleton became progressively depolarized with patches spread around the cell cortex, which was concurrent with loss of cylindrical morphology (Figures 1A and 6B and data not shown) and further sterol spreading. Thus, total depolarized growth is preceded by a defect in NETO arising from defective localization of F-actin at the new cell end.

### btn1Δ cells are defective in endocytosis

We then examined *btn1*Δ cells grown at 37°C for defects in endocytosis 30). Severely reduced uptake of FM4-64 was observed in *btn1*Δ cells at 18 h at 37°C (Figure 6E). This was consistent with total depolarization of F-actin and sterol domains (Figure 6E). FM4-64 uptake was also severely reduced at 7 h when F-actin defects are apparent and sterol spreading is first observed. Upon examination of the localization of FM4-64 that had been taken up, we saw that in contrast to wild-type cells, which showed typical staining of cytoplasmic structures, FM4-64 was retained at one cell pole in interphase cells and no obvious vacuole staining was observed (Figure 6F), suggesting a defect both in uptake and in trafficking to the vacuole. Interestingly, uptake of FM4-64 was also reduced in *btn1*Δ cells that have been grown for 4 h at 37°C (Figure 6E). This is consistent with a mild defect in F-actin, visualized by slightly diffused actin staining, at this time-point (Figure 6C) but prior to sterol spreading (Figure 2C). This suggests that in *btn1*Δ cells, sterol spreading may be a downstream consequence of defects in F-actin patch formation/polarization and endocytosis. This is consistent with our observations that Latrunculin A treatment, which causes a total block in endocytosis 33), is followed by sterol spreading even in wild-type cells (Figure 4).

As previously reported 16), uptake and trafficking of FM4-64 were relatively normal in *btn1*Δ cells at the permissive temperature of 25°C when F-actin patches and sterol domains were largely polarized (Figures 2A and 6A), although some diffuse actin was evident and uptake of FM4-64 is slightly reduced over time (Figure 5A,B). This implies that at 25°C and under normal laboratory growth conditions, Btn1p plays a minor role in F-actin patch remodelling and endocytosis (as monitored by FM4-64 uptake) but has little effect on sterol domains, but at 37°C, this role is crucial.

### Btn1p has a polarized location at 37°C

**Figure 7:**
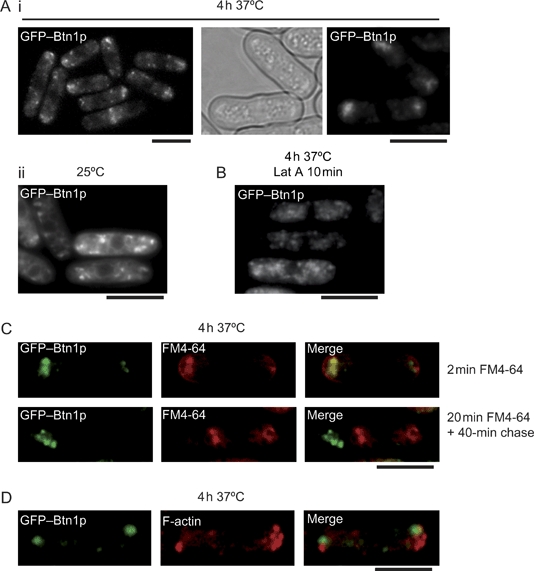
**Btn1p has an altered intracellular distribution at 37°C.**A) Polarized location of GFP–Btn1p at 37°C: location of GFP–Btn1p in wild-type ED665 cells after overnight induction at 25°C followed by growth for 4 h at i) 37°C or ii) 25°C. B) Cell tip localization of GFP–Btn1p is actin dependent: wild-type ED665 cells expressing GFP–Btn1p at 4 h at 37°C treated with 10 μm Latrunculin A (Lat A) for 10 min at 37°C. C) GFP–Btn1p colocalizes with early but not late FM4-64-staining vesicles near cell tips at 37°C: 2 min FM4-64 uptake in wild-type ED665 cells expressing GFP–Btn1p at 4 h at 37°C (upper panel) and 20 min FM4-64 uptake followed by 40-min chase in wild-type ED665 cells expressing GFP–Btn1p grown at 37°C for 4 h (including labelling time) (lower panel). D) GFP–Btn1p localizes adjacent to, or partially overlapping, polarized F-actin patches: wild-type ED665 cells expressing GFP–Btn1p at 37°C for 4 h were fixed and stained for actin with rhodamine–phalloidin. Scale bar, 10 μm.

For Btn1p to affect events that normally occur at the cell poles or septum, that is, the formation and polarization of F-actin patches, endocytosis, polarization of sterol-rich domains and cell wall deposition, it might be expected that its location be spatially regulated, perhaps at these sites. We have previously shown that trafficking of Btn1p to the vacuole was through the endocytic pathway and was slower than for other vacuole membrane proteins and that it exerts an effect prior to reaching the vacuole 16), consistent with a prevacuolar location of Btn1p being functionally important. Because cells lacking *btn1* have additional and severe defects when grown at 37°C, we examined the intracellular location of Btn1p at this temperature and found that it was altered. GFP–Btn1p was enriched at the cell poles and at the septa in wild-type cells grown at 37°C (Figure 7A) compared with cells grown at 25°C where it was located in structures distributed throughout the cytoplasm (Figure 7A) 16). The polar location of GFP–Btn1p was shown to be actin dependent as treatment with Latrunculin A, which prevents polymerization of actin, but not with methyl benzimidazol-2-yl carbamate (MBC), which depolymerizes microtubules, caused Btn1p to disperse throughout the cell (Figure 7B and data not shown). Importantly, the location of GFP–Btn1p in cells grown at 37°C overlapped that of early FM4-64-staining vesicles at the cell poles, but not late FM4-64-staining vacuoles, indicating that these Btn1p-containing compartments are prevacuolar and within the endomembrane system (Figure 7C). Intriguingly, the GFP–Btn1p-containing structures were found to localize adjacent to F-actin patches near the cell pole regions (Figure 7D). Thus, the location and trafficking of Btn1p appear to be differentially regulated at high temperature, with Btn1p being retained in compartments that locate at the cell poles at 37°C. Because Btn1p does not completely colocalize with F-actin patches at the plasma membrane, this suggests that Btn1p may be acting from a separate compartment that impacts on the early endocytic/F-actin patch machinery.

### Btn1p is required for bipolar growth at 37°C

**Figure 8:**
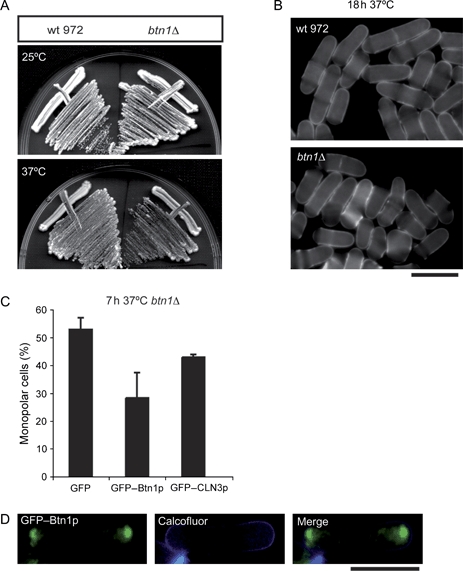
**Btn1p is required for bipolar growth at 37°C.**A) Wild-type 972 and *btn1*Δ cells grown on MM agar at 25°C or 37°C for 5 days. B) *btn1* Δ cells have a monopolar growth defect in MM after growth at 37°C: calcofluor images of indicated cells grown at 37°C for 18 h. C) Expression of GFP–Btn1p restores bipolar growth in *btn1*Δ cells at 7 h at 37°C in MM: graph showing the number of monopolar cells as a percentage of the total population of *btn1*Δ cells expressing GFP, GFP–Btn1p or human GFP–CLN3p. D) Polarized location of GFP–Btn1p in *btn1*Δ cells grown at 37°C for 7 h with calcofluor staining. Scale bar, 10 μm.

We have previously shown that ectopic expression of Btn1p rescues the cytokinesis defect displayed by *btn1*Δ cells grown at 25°C in minimal medium (MM) 16). Surprisingly, the depolarized phenotype and lysis of *btn1*Δ cells were not as severe when these cells were grown at 37°C in MM, a minimal media used to select expression plasmids (Figure 8A). Although minimal cell swelling or lysis was apparent upon growth in MM, we found that these cells still retained a NETO defect as evidenced by monopolar growth (Figure 8B) because at 7 h at 37°C, 53% of septated *btn1*Δ cells were monopolar for growth (Figure 8C). We examined whether expression of Btn1p and CLN3 could rescue this defect. We found that ectopic expression of GFP–Btn1p can rescue the monopolar growth phenotype and restore bipolar growth in *btn1*Δ cells (Figure 8C,D) and expression of CLN3 can partially restore bipolar growth (Figure 8C). We have previously shown that CLN3 traffics faster to the vacuole in *btn1*Δ cells at 25°C 16). Thus, the prolonged prevacuolar location of ectopically expressed Btn1p may be functionally important in establishing/maintaining polarized growth, further supporting its involvement in a pathway that affects F-actin patch formation, endocytosis and location of sterol-rich domains that is severely exacerbated in response to heat stress.

## Discussion

*CLN3* was first identified as the gene underlying JNCL in 1995 36), and recent reports have suggested multiple and apparently unconnected phenotypes associated with defects in its function. Despite much effort, its mechanism of action is still unknown, although its conservation suggests that it has a basic cellular role. Our results using the fission yeast *Sz. pombe* shed further light on the apparent complexity of *CLN3* function. We describe several phenotypes associated with loss of *btn1*, the *Sz. pombe* orthologue of *CLN3*, and investigation of the underlying molecular pathways provided evidence of a link between these and with Btn1p function (Table 1).

**Table 1:** Summary of phenotypes of *myo1*Δ, *end4*Δ and *btn1*Δ cells at 25°C and of *btn1*Δ cells at 37°C

Phenotype^a^	*btn1*Δ at 25°C	*myo1*Δ at 25°C	*end4*Δ at 25°C	*btn1*Δ after 18 h at 37°C
Cell lysis	−	+	+	++
Depolarized sterols	−	++	++	++
Depolarized F-actin	+	++	++	++
Endocytosis defects	+	++	++	++
Depolarized growth	−	++	++	++

a These phenotypes are absent in wild-type cells; + is mild, ++ is severe, − is no defect.

We discovered that Btn1p is essential for polarized growth, particularly at high temperature. Progressive depolarization and spreading of sterol-rich domains commenced after the first cell cycle (7 h) at 37°C, and this was preceded by a progressive failure in the formation and polarization of F-actin patches and by endocytic defects that we now show are linked. Previously, sterol domains and F-actin patch polarization have separately been linked to Myo1p (19,20,21), and consistent with this, we found a loss of polarized location of Myo1p in *btn1*Δ cells after 7 h at the restrictive temperature. We additionally show that Myo1p is required for efficient endocytosis, and we directly link endocytosis with polarization of sterol-rich domains because *end4*Δ cells, which are severely compromised in endocytosis, exhibit total sterol spreading. *end4* Δ cells (32,37), like *btn1*Δ cells, have a block in NETO prior to total depolarization of growth. Thus, in *Sz. pombe*, the formation and polarization of F-actin patches and the early stages of endocytosis are intimately linked with the polarization of sterol-rich domains, and co-ordination of these aspects is required both for establishing new sites for growth (NETO) and for maintaining polarized and rod-shaped growth. We speculate that under certain conditions, defects in the positioning of the early endocytic vesicle/F-actin patch formation machinery during NETO give rise to sterol spreading and a feedback system of further depolarized growth, resulting from a loss of co-ordination of the endocytic and secretory pathways that specify sites for delivery of membrane and new cell wall components. We further speculate that the process of endocytosis regulates and delimits these sites to the cell tips/septum and that a major purpose of endocytosis in *Sz. pombe* is to co-ordinate membrane retrieval from the surface during ongoing polarized secretion.

We had previously observed an underlying cell cycle delay at 25°C in *btn1*Δ cells in addition to the cytokinesis defect with longer and more binucleate cells 16). These cell cycle delays could be because of a delay in remodelling of the polarization and actin-dependent machinery at each major transition throughout the cell cycle, in particular during NETO. Exploration of the phenotypes of *btn1*Δ cells under normal growth conditions (25°C) revealed that the uptake and trafficking of FM4-64 are slightly reduced in these cells at the permissive temperature of 25°C when Myo1p and sterol domains appear to be polarized, although F-actin patches are slightly abnormal, consistent with Btn1p playing a minor role in endocytosis (as monitored by FM4-64 uptake) at 25°C and under normal growth conditions. Further investigation is required to fully elucidate the role that *btn1* plays in these processes under non-stress conditions.

While the link between F-actin patches, the early steps of endocytic membrane invagination and establishment of polarity is well understood in *S. cerevisiae*^30^) and to a lesser extent in *Sz. pombe*^33^), this is only just being explored in mammalian systems (38–40). It is tempting to speculate that an intimate link exists between F-actin, endocytosis, the type 1 myosins and sterol polarization across all eukaryotes. Furthermore, from our data in the *Sz. pombe* system, we propose that F-actin-dependent early endocytic events are upstream of sterol and Myo1p-related polarity events.

Mammalian cells have eight type 1 myosins, two of which (MYO1E and MYO1F) are long-tailed like *Sz. pombe* Myo1p. MYO1E plays a role in endocytosis 41), like Myo1p, and MYO1F is important for cell migration 42). It will be interesting to investigate whether the function of Btn1p in relation to polarity, cytoskeletal regulation, endocytosis, Myo1p, and sterols, is conserved through CLN3 in higher eukaryotes. Certainly, endocytic defects have been reported in mammalian systems with *CLN3* mutations (8,9). A similar depolarization phenotype has not been reported for *S. cerevisiae* lacking the orthologue *BTN1* despite varying growth, temperature and hyperosmotic conditions (43,44). This may be a reflection of some important difference in their biology or the presence in *S. cerevisiae* of two long-tailed type 1 myosins that functionally compensate for each other 31). *S. cerevisiae* cells also differ from *Sz. pombe* in their requirement for a programme of polarization only during budding of a new daughter cell. *Sz. pombe*, in contrast, relies on a continuous programme of polarized cell growth to maintain and restrict growth to the cell tips. It will be interesting to check for polarization of bud formation in the mother cell and its daughters in *S. cerevisiae* deleted for *btn1*, particularly following heat or other stress.

An important question raised by this data is how a protein that is considered to be a vacuole/lysosome membrane protein impacts on early endocytosis, cytoskeletal dynamics and the polarity machinery (including sterol-rich domains). We have previously provided evidence that Btn1p traffics slowly to the vacuole through the endocytic system and that Btn1p has a function prior to reaching the vacuole 16). We now show that the location of Btn1p within endomembrane compartments is controlled, particularly in response to heat stress, when the location of Btn1p becomes polarized in an actin-dependent manner at sites of active growth. We propose that it is from these endomembrane compartments that Btn1p is affecting the F-actin machinery and sterol domains. Interestingly, CLN3 has been found to have additional polarized location in neurons where it localizes at the synapse (5,7,45,46). Thus, the evidence suggests that Btn1p and also CLN3 are spatially controlled and that their main function is outside the vacuole/lysosome.

In addition to the novel phenotypes reported in this paper, Btn1p and CLN3 play a role in vacuole/lysosome acidification (10,16,47,48) under normal conditions. Vacuole/lysosome acidification by v-ATPase is modulated by many factors, including the actin cytoskeleton (49–57); so, Btn1p may be affecting vacuole pH by indirectly modulating v-ATPase activity by an effect on the cytoskeleton. Alternatively, or in addition, Btn1p may be affecting the trafficking of v-ATPase and perhaps other transporters to the vacuole, again through its effect on actin.

In conclusion, we have further characterized the phenotype of cells deleted for the *Sz. pombe* orthologue of the human Batten disease gene *CLN3*, *btn1*. We identified a failure to correctly polarize F-actin patches and the endocytic machinery, leading to the loss of polarity of Myo1p and sterol-rich membrane domains. Our work suggests that CLN3 may act in a similar manner and affect the regulation of the actin cytoskeleton and membrane trafficking, perhaps resulting in changes in lipid domains. This could have far-reaching consequences on the trafficking and activity of other proteins including CLN3 itself as well as type 1 myosins. Failure to correctly regulate the endocytic pathway could give rise to many of the features of JNCL, for example, defective antigen processing, leading to the production of autoantibodies (58,59) or aberrant modulation of receptor signalling in neurons leading to seizures 60). The precise events that contribute to neuronal cell death in JNCL remain to be delineated. We are continuing to use the *Sz. pombe* system to investigate the basic function of Btn1p, and by extrapolation CLN3, and expect this to provide new avenues for the development of therapies for patients with JNCL.

## Materials and Methods

### Yeast strains and general techniques

Strains used in this study are listed in Table 2. Media, growth, maintenance of strains and genetic methods were as described 61). Cells were grown in rich medium (YES) or synthetic ‘minimal’ medium (MM) containing appropriate supplements. For protein expression, cells were grown overnight in MM plus thiamine (4 μm). Cells were washed three times in MM lacking thiamine and grown for 18 h in the same medium. Latrunculin A (Molecular probes) was used at a final concentration of 10 μm and MBC (Sigma) at a final concentration of 25 μm.

**Table 2:** Strains

Strain	Genotype	Source
972	*h*^−^	Laboratory stock
ED665	*h*^−^*, ura4-D18, leu1-32, his2*	Laboratory stock
YG660 (*btn1*Δ)	*h^+^, btn1::leu2, ura4-D18, leu1-32, his2*	Laboratory stock
SC2D (*btn1*Δ)	*h*^−^*, btn1::leu2, leu1-32*	Laboratory stock
SC36A (ED665 + pREP42GFPBtn1)	*h*^−^*, ura4-D18, leu1-32, his2*	Laboratory stock
SC1A (YG660 + pREP42GFP)	*h^+^, btn1::leu2, ura4-D18, leu1-32, his2*	Laboratory stock
SC4A (YG660 + pREP42GFPBtn1)	*h^+^, btn1::leu2, ura4-D18, leu1-32, his2*	Laboratory stock
SC31A (YG660 + pREP42GFPCLN3)	*h^+^, btn1::leu2, ura4-D18, leu1-32, his2*	Laboratory stock
*myo1*Δ (TP190)	*h*^−^*, myo1::kanMX, leu1-32, ura4-D18, ade6, his3-D1*	T. D. Pollard
*GFP-myo1*	*h*^−^*, kanMX-Pmyo1-mGFP-myo1, leu1-32, his-D1, ura4-D18, ade6-M216*	T. D. Pollard
SC505D (*btn1*Δ*GFP-myo1*)	*h*^−^*, kanMX-Pmyo1-mGFP-myo1, btn1::leu2, leu1-32, his-D1, his2, ura4-D18*	This study
*sla2/end4*Δ	*h*^−^*end4::kanMX leu1-32, ura4-D18, ade6-M210*	S. Castagnetti
*cps8-188*	*H, cps8-188, ura4-D18*	W. Kobayashi

### Fluorescence staining and microscopy

For cell fixation, cells in log-phase growth were fixed in 10% formaldehyde for 15 min, washed three times in ×1 PBS and stored at 4°C. Calcofluor (50 μg/mL; Polysciences Inc.) was used to visualize septa and cell wall deposition 61). Cells were stained with 4′,6-diamidino-2-phenylindole (DAPI) for DNA. For endocytosis assays, FM4-64 dye (Molecular Probes) was dissolved in dimethyl sulphoxide at a concentration of 0.82 mm. Typically, 2 μL FM4-64 stock was added to 1 mL cells at equal cell density, typically 2.5 × 10^6^ cell/mL (1.64 μm final concentration), and cells incubated at 25°C for 20 min and where indicated chased for 40 min in fresh media to allow all FM4-64 to reach the vacuole. Samples were viewed immediately. In one experiment, excess FM4-64 was preloaded onto cells on ice 19) prior to uptake. For FM4-64 densitometry measurements, 20 μL FM4-64 was added to 10 mL cells, and cells were grown at 25°C or 37°C with shaking. At indicated times (typically 5, 10, 15, 20 and 60 min), 1 mL of cells were removed, washed in fresh, cold media to remove FM4-64 that had not been taken up and stored on ice until image capture. For densitometry measurements, *n* = 30 individual cell measurements were obtained using openlab software. For visualization of sterol-rich domains, filipin staining was performed as described 18) and at a final concentration of 5 μg/mL. Briefly, 1 μL of filipin was added to 1 mL log-phase cells (at equal cell density, typically 2.5 × 10^6^ cell/mL), and cells were rapidly washed in media and visualized immediately. All cells (wild type or mutant) were kept at the stated temperature until just prior to image capture and were treated identically. Microscopy (at room temperature) was rapidly performed to minimize any changes in temperature. Measurements were obtained using openlab software from *n* = 25 cells for interphase measurements and *n* = 25 for septated cell measurements and transferred to Excel for analysis. The mean fluorescence intensity of a set tool area was obtained in the same cell for three non-overlapping regions – cell tip, cell septum (if present) and cell side (which was equidistant between the mid region and the tip of the cell). For cells at 37°C, the same set area was used. Septation in depolarized cells gave the orientation. Most depolarized interphase cells retained an elliptical or egg shape, allowing orientation for positioning the measuring tool. Cells with septa that had not separated were considered septated cells. For actin staining, cells were fixed by adding formaldehyde (to a final concentration of 3.7%) to the culture for 60 min with shaking, washed twice with PM (35 mm K_2_HPO_4_ and 0.5 mm MgSO_4_, pH 6.9), permeabilized with 1% Triton-X-100, washed twice with PM and stained with rhodamine–phalloidin (Molecular Probes). We note that the defects present in *btn1*Δ cells may interfere with visualization of intracellular structures. Images were visualized using a Hamamatsu digital camera C4742-95 fitted to a Zeiss Axioskop microscope with plan-Apochromat ×63 1.25 oil objective and were recorded using openlab 3.4 software (Improvision Ltd.) and downloaded to either Microsoft Excel for analysis or to adobe photoshop 7 for assembly into montages. A DAPI filter was used for DAPI, calcofluor and filipin fluorescence. A fluorescein isothiocyanate filter was used for GFP detection and a rhodamine filter for detection of FM4-64.

## Supplementary material

Figure S1: Absence of actin staining following Latrunculin A treatment. Rhodamine-phalloidin stain for actin in wild-type (wt) cells (contr) and the same cells treated with Latrunculin A for 2 and 4 h. Scale bar, 10 μm.

Figure S2: myo1▵ cells are delayed in uptake of FM4-64 by endocytosis. FM4-64 uptake experiments were performed using the protocol described by Takeda and Chang (19). Cells were loaded with 20 μM FM4-64 from 2 mM stock for 15 min on ice, washed once with ice-cold YES, resuspended in fresh YES, incubated at 30°C and FM4-64 imaged at different time-points. The figure shows indicated cells at time 0 (i.e. after15-min incubation on ice and washing) and at 2, 5, 10 and 20 min after release into fresh media at the raised temperature. In wild-type (wt) cells, FM4-64 is internalized rapidly (by 2 min) and has reached the vacuole in some cells by 5 min and in all cells by 20 min. In contrast, in myo1▵ cells, FM4-64 remains at the cell surface for longer and internalization is much slower. FM4-64 is only evident at the vacuole in some cells at 20 min. Scale bar, 10 μm.

## References

[1] Janes RW, Munroe PB, Mitchsion HM, Gardiner RM, Mole SE, Wallace BA (1996). A model for Batten disease protein CLN3: functional implications from homology and mutations. FEBS Lett.

[2] Mao Q, Foster BJ, Xia H, Davidson BL (2003). Membrane topology of CLN3, the protein underlying Batten disease. FEBS Lett.

[3] Ezaki J, Takeda-Ezaki M, Koike M, Ohsawa Y, Taka H, Mineki R, Murayama K, Uchiyama Y, Ueno T, Kominami E (2003). Characterization of Cln3p, the gene product responsible for juvenile neuronal ceroid lipofuscinosis, as a lysosomal integral membrane glycoprotein. J Neurochem.

[4] Kyttälä A, Ihrke G, Vesa J, Schell MJ, Luzio JP (2004). Two motifs target Batten disease protein CLN3 to lysosomes in transfected non-neuronal and neuronal cells. Mol Biol Cell.

[5] Storch S, Pohl S, Quitsch A, Falley K, Braulke T (2007). C-terminal prenylation of the CLN3 membrane glycoprotein is required for efficient endosomal sorting to lysosomes. Traffic.

[6] Rakheja D, Narayan SB, Pastor JV, Bennett MJ (2004). CLN3P, the Batten disease protein, localizes to membrane lipid rafts (detergent-resistant membranes). Biochem Biophys Res Commun.

[7] Järvelä I, Lehtovirta M, Tikkanen R, Kyttälä A, Jalanko A (1999). Defective intracellular transport of CLN3 is the molecular basis of Batten disease (JNCL). Hum Mol Genet.

[8] Fossale E, Wolf P, Espinola JA, Lubicz-Nawrocka T, Teed AM, Gao H, Rigamonti D, Cattaneo E, MacDonald ME, Cotman SL (2004). Membrane trafficking and mitochondrial abnormalities precede subunit c deposition in a cerebellar cell model of juvenile neuronal ceroid lipofuscinosis. BMC Neurosci.

[9] Luiro K, Yliannala K, Ahtiainen L, Maunu H, Järvelä I, Kyttala A, Jalanko A (2004). Interconnections of CLN3, Hook1 and Rab proteins link Batten disease to defects in the endocytic pathway. Hum Mol Genet.

[10] Holopainen JM, Saarikoski J, Kinnunen PK, Järvelä I (2001). Elevated lysosomal pH in neuronal ceroid lipofuscinoses (NCLs). Eur J Biochem.

[11] Ramirez-Montealegre D, Pearce DA (2005). Defective lysosomal arginine transport in juvenile Batten disease. Hum Mol Genet.

[12] Cao Y, Espinola JA, Fossale E, Massey AC, Cuervo AM, Macdonald ME, Cotman SL (2006). Autophagy is disrupted in a knock-in mouse model of juvenile neuronal ceroid lipofuscinosis. J Biol Chem.

[13] Persaud-Sawin DA, VanDongen A, Boustany RM (2002). Motifs within the CLN3 protein: modulation of cell growth rates and apoptosis. Hum Mol Genet.

[14] Narayan SB, Rakheja D, Tan L, Pastor JV, Bennett MJ (2006). CLN3P, the Batten's disease protein, is a novel palmitoyl-protein Delta-9 desaturase. Ann Neurol.

[15] Hobert JA, Dawson G (2007). A novel role of the Batten disease gene CLN3: association with BMP synthesis. Biochem Biophys Res Commun.

[16] Gachet Y, Codlin S, Hyams JS, Mole SE (2005). *btn1*, the fission yeast homologue of the human Batten disease gene, *CLN3*, regulates vacuole homeostasis. J Cell Sci.

[17] Degols G, Shiozaki K, Russell P (1996). Activation and regulation of the Spc1 stress-activated protein kinase in *Schizosaccharomyces pombe*. Mol Cell Biol.

[18] Wachtler V, Rajagopalan S, Balasubramanian MK (2003). Sterol-rich plasma membrane domains in the fission yeast *Schizosaccharomyces pombe*. J Cell Sci.

[19] Takeda T, Chang F (2005). Role of fission yeast myosin I in organization of sterol-rich membrane domains. Curr Biol.

[20] Lee WL, Bezanilla M, Pollard TD (2000). Fission yeast myosin-I, Myo1p, stimulates actin assembly by Arp2/3 complex and shares functions with WASp. J Cell Biol.

[21] Sirotkin V, Beltzner CC, Marchand JB, Pollard TD (2005). Interactions of WASp, myosin-I, and verprolin with Arp2/3 complex during actin patch assembly in fission yeast. J Cell Biol.

[22] Sun Y, Martin AC, Drubin DG (2006). Endocytic internalization in budding yeast requires coordinated actin nucleation and myosin motor activity. Dev Cell.

[23] Soulard A, Lechler T, Spiridonov V, Shevchenko A, Shevchenko A, Li R, Winsor B (2002). *Saccharomyces cerevisiae* Bzz1p is implicated with type I myosins in actin patch polarization and is able to recruit actin-polymerizing machinery in vitro. Mol Cell Biol.

[24] Soulard A, Friant S, Fitterer C, Orange C, Kaneva G, Mirey G, Winsor B (2005). The WASP/Las17p-interacting protein Bzz1p functions with Myo5p in an early stage of endocytosis. Protoplasma.

[25] D'Agostino JL, Goode BL (2005). Dissection of Arp2/3 complex actin nucleation mechanism and distinct roles for its nucleation-promoting factors in *Saccharomyces cerevisiae*. Genetics.

[26] Jonsdottir GA, Li R (2004). Dynamics of yeast Myosin I: evidence for a possible role in scission of endocytic vesicles. Curr Biol.

[27] Lees-Miller JP, Henry G, Helfman DM (1992). Identification of *act2*, an essential gene in the fission yeast *Schizosaccharomyces pombe* that encodes a protein related to actin. Proc Natl Acad Sci U S A.

[28] Morrell JL, Morphew M, Gould KL (1999). A mutant of Arp2p causes partial disassembly of the Arp2/3 complex and loss of cortical actin function in fission yeast. Mol Biol Cell.

[29] Ishiguro J, Kobayashi W (1996). An actin point-mutation neighboring the ‘hydrophobic plug’ causes defects in the maintenance of cell polarity and septum organization in the fission yeast Schizosaccharomyces pombe. FEBS Lett.

[30] Kaksonen M, Toret CP, Drubin DG (2006). Harnessing actin dynamics for clathrin-mediated endocytosis. Nat Rev Mol Cell Biol.

[31] Goodson HV, Anderson BL, Warrick HM, Pon LA, Spudich JA (1996). Synthetic lethality screen identifies a novel yeast myosin I gene (MYO5): myosin I proteins are required for polarization of the actin cytoskeleton. J Cell Biol.

[32] Iwaki T, Tanaka N, Takagi H, Giga-Hama Y, Takegawa K (2004). Characterization of *end4+*, a gene required for endocytosis in *Schizosaccharomyces pombe*. Yeast.

[33] Gachet Y, Hyams JS (2005). Endocytosis in fission yeast is spatially associated with the actin cytoskeleton during polarised cell growth and cytokinesis. J Cell Sci.

[34] Rupes I, Jia Z, Young PG (1999). Ssp1 promotes actin depolymerization and is involved in stress response and new end take-off control in fission yeast. Mol Biol Cell.

[35] Martin SG, Chang F (2005). New end take off: regulating cell polarity during the fission yeast cell cycle. Cell Cycle.

[36] The International Batten Disease Consortium (1995). Isolation of a novel gene underlying Batten disease, *CLN3*. Cell.

[37] Castagnetti S, Behrens R, Nurse P (2005). End4/Sla2 is involved in establishment of a new growth zone in *Schizosaccharomyces pombe*. J Cell Sci.

[38] Balklava Z, Pant S, Fares H, Grant BD (2007). Genome-wide analysis identifies a general requirement for polarity proteins in endocytic traffic. Nat Cell Biol.

[39] Smythe E, Ayscough KR (2006). Actin regulation in endocytosis. J Cell Sci.

[40] Chadda R, Howes MT, Plowman SJ, Hancock JF, Parton RG, Mayor S (2007). Cholesterol-sensitive Cdc42 activation regulates actin polymerization for endocytosis via the GEEC pathway. Traffic.

[41] Krendel M, Osterweil EK, Mooseker MS (2007). Myosin 1E interacts with synaptojanin-1 and dynamin and is involved in endocytosis. FEBS Lett.

[42] Kim SV, Mehal WZ, Dong X, Heinrich V, Pypaert M, Mellman I, Dembo M, Mooseker MS, Wu D, Flavell RA (2006). Modulation of cell adhesion and motility in the immune system by Myo1f. Science.

[43] Pearce DA, Sherman F (1998). A yeast model for the study of Batten disease. Proc Natl Acad Sci USA.

[44] Croopnick JB, Choi HC, Mueller DM (1998). The subcellular location of the yeast *Saccharomyces cerevisiae* homologue of the protein defective in the juvenile form of Batten disease. Biochem Biophys Res Commun.

[45] Kremmidiotis G, Lensink IL, Bilton RL, Woollatt E, Chataway TK, Sutherland GR, Callen DF (1999). The Batten disease gene product (CLN3p) is a Golgi integral membrane protein. Hum Mol Genet.

[46] Luiro K, Kopra O, Lehtovirta M, Jalanko A (2001). CLN3 protein is targeted to neuronal synapses but excluded from synaptic vesicles: new clues to Batten disease. Hum Mol Genet.

[47] Pearce DA, Ferea T, Nosel SA, Das B, Sherman F (1999). Action of *BTN1*, the yeast orthologue of the gene mutated in Batten disease. Nat Genet.

[48] Padilla-Lopez S, Pearce DA (2006). *Saccharomyces cerevisiae* lacking Btn1p modulate vacuolar ATPase activity in order to regulate pH imbalance in the vacuole. J Biol Chem.

[49] Holliday LS, Lu M, Lee BS, Nelson RD, Solivan S, Zhang L, Gluck SL (2000). The amino-terminal domain of the B subunit of vacuolar H+-ATPase contains a filamentous actin binding site. J Biol Chem.

[50] Vitavska O, Wieczorek H, Merzendorfer H (2003). A novel role for subunit C in mediating binding of the H+-V-ATPase to the actin cytoskeleton. J Biol Chem.

[51] Vitavska O, Merzendorfer H, Wieczorek H (2005). The V-ATPase subunit C binds to polymeric F-actin as well as to monomeric G-actin and induces cross-linking of actin filaments. J Biol Chem.

[52] Zuo J, Jiang J, Chen SH, Vergara S, Gong Y, Xue J, Huang H, Kaku M, Holliday LS (2006). Actin binding activity of subunit B of vacuolar H+-ATPase is involved in its targeting to ruffled membranes of osteoclasts. J Bone Miner Res.

[53] Phillips MD, Thomas GH (2006). Brush border spectrin is required for early endosome recycling in Drosophila. J Cell Sci.

[54] Lee BS, Gluck SL, Holliday LS (1999). Interaction between vacuolar H(+)-ATPase and microfilaments during osteoclast activation. J Biol Chem.

[55] Beaulieu V, Da Silva N, Pastor-Soler N, Brown CR, Smith PJ, Brown D, Breton S (2005). Modulation of the actin cytoskeleton via gelsolin regulates vacuolar H+-ATPase recycling. J Biol Chem.

[56] Okumura S, Mizoguchi T, Sato N, Yamaki M, Kobayashi Y, Yamauchi H, Ozawa H, Udagawa N, Takahashi N (2006). Coordination of microtubules and the actin cytoskeleton is important in osteoclast function, but calcitonin disrupts sealing zones without affecting microtubule networks. Bone.

[57] Drory O, Nelson N (2006). Structural and functional features of yeast V-ATPase subunit C. Biochim Biophys Acta.

[58] Chattopadhyay S, Ito M, Cooper JD, Brooks AI, Curran TM, Powers JM, Pearce DA (2002). An autoantibody inhibitory to glutamic acid decarboxylase in the neurodegenerative disorder Batten disease. Hum Mol Genet.

[59] Lim MJ, Alexander N, Benedict JW, Chattopadhyay S, Shemilt SJ, Guerin CJ, Cooper JD, Pearce DA (2007). IgG entry and deposition are components of the neuroimmune response in Batten disease. Neurobiol Dis.

[60] Michels G, Moss SJ (2007). GABAA receptors: properties and trafficking. Crit Rev Biochem Mol Biol.

[61] Moreno S, Klar A, Nurse P (1991). Molecular genetic analysis of fission yeast *Schizosaccharomyces pombe*. Methods Enzymol.

